# Host-specific growth responses of *Larix kaempferi* and *Quercus acutissima* to Asian gypsy moth defoliation in central Korea

**DOI:** 10.1038/s41598-024-51907-w

**Published:** 2024-01-17

**Authors:** Jong Bin Jung, Eun-Sook Kim, Jong-Hwan Lim, Won Il Choi

**Affiliations:** Forest Ecology Division, National Institute of Forest Science, 57 Hoegi-Ro, Dongdaemun-Gu, Seoul, 02455 Republic of Korea

**Keywords:** Ecology, Environmental sciences

## Abstract

As the risk of gypsy moth outbreaks that have detrimental effects on forest ecosystem in the Northern Hemisphere increase due to climate change, a quantitative evaluation of the impact of gypsy moth defoliation is needed to support the adaptive forest management. To evaluate the host-specific impact of gypsy moth defoliation, radial growth and annual carbon accumulation were compared for one severely defoliated (*Larix kaempferi* (Lamb.) Carrière) and one moderate defoliated (*Quercus acutissima* Carruth.) host, in defoliated and non-defoliated site using tree-ring analysis. Finally, the resilience indices of radial growth variables were calculated to assess the ability of sampled trees to withstand defoliation. Gypsy moth defoliation mainly decreased latewood width and caused reduction in annual carbon absorption more than 40% for both tree species. However, *L. kaempferi,* showed the reduced growth until the year following defoliation, while *Q. acutissima,* showed no lagged growth depression and rapid growth recover. The findings show how each species reacts differently to gypsy moth defoliation and highlight the need of managing forests in a way that takes resilient tree species into account.

## Introduction

Outbreaks of insect pests are a natural disturbance, which may cause economic and ecological impacts by changing the structure and function of forest ecosystems^1^. Massive outbreaks of insect pests that affect the growth of host trees may increase tree mortality, causing a reduction of forest carbon storage^2,3^. For example, extensive outbreak of mountain pine beetle (*Dendroctonus ponderosae* Hopkins) in coniferous forests in British Columbia, Canada, changed the forests from carbon sinks to sources^4^. Under the influence of climate change, it is expected that large insect outbreaks will become more frequent and severe in the future, and the resulting loss of forest function is likely to also increase^5^. Detecting and evaluating the damage caused by insect outbreaks are essential for managing and mitigating their impact on forest functioning in a changing environment.

The gypsy moth (*Lymantria dispar* L.), which is native to Eurasia, is one of the most widespread and polyphagous defoliators that have detrimental effects on forest ecosystem^6^. Among its subspecies, European gypsy moth (EGM) has been a concern for over a century due to its rapid spread in North America^7^. Trees in genera *Quercus*, *Populus*, and *Salix* are known to be the most preferred hosts for this subspecies, and the eco-physiological impacts of EGM defoliation have been extensively investigated^8,9^. Asian gypsy moth (AGM) in Far East Asia also has a broad host spectrum, including conifer species such as *Larix* spp. and various deciduous species in the genera *Quercus*, *Fagus* and *Betula*^10–12^. However, the impact of AGM defoliation has received limited attention than EGM, despite its capacity to cause damage to numerous hosts. Particularly in Korea, gypsy moth is regarded as a sporadic pest, likely due to the regulation of populations by natural enemies^13^. Nonetheless, there have been periodic outbreaks in Japan and Russia^10,14^, with intensive defoliation having a negative impact on the growth and reproduction of multiple hosts^11,15^.

Gypsy moth defoliation causes multi-level effects, from impacts within tree tissues (xylogenesis) to changes in ecosystem functioning^8^. The larvae hatch in spring and actively feed on foliage during early- and mid-summer, which results in insufficient carbohydrate accumulation for xylem formation^16^. Many studies have reported that damage by spring defoliators cause trees to produce abnormal cells in the latewood that have small lumens and thin cell walls. This effect leads to growth rings with narrow latewood areas, the so-called light ring, which typical of wood formed after spring defoliation^17,18^. Defoliation also reduces the number of cells in tree-rings, leading to radial growth reduction during or after the defoliation^19,20^. This decrease of radial growth results in an overall decline in forest productivity, and eventually can affect the forest’s carbon dynamics^21,22^.

The impact of defoliation is host species-dependent and seems to be related to host preference of gypsy moth^9,23^. For EGM, the radial growth loss in preferred species such as *Quercus rubra* L. and *Populus grandidentat*a Michx. was higher than that in the non-preferred species^20^. Some other species such as *Fraxinus americana* L. and *Pinus strobus* L. were rarely defoliated and thus suffered little from outbreaks^9,20,24^. However, this correlation between proportional reduction of radial growth and host preference has not always been observed. For example, *Populus tremuloides* Michx., which is another highly preferred host for EGM, showed no or little change in radial growth after defoliation^20^. Similarly, EGM caused severe damage to a resistant species, *Pinus radiata* D. Don, in northwestern Spain, with a radial growth reduction of up to 74%^25^. These findings emphasized the need for study on the host-specific growth response to AGM to better understand the impact of gypsy moth defoliation.

Despite being native to the Korean Peninsula, the gypsy moth (AGM) outbreaks were rarely reported^26,27^. However, a recent nationwide gypsy moth outbreak occurred in Korea that severely damaged about 4300 ha of forest, raising concerns over the effects of defoliation on forest ecosystems^28^. While their feeding preferences are not well-known, a recent study identified 46 species of coniferous and broad-leaved species suffering from severe to low canopy feeding damage^28^. Such defoliation of the gypsy moth could alter forest structure and composition in Korea and affect forest functions by damaging multiple hosts.

Our goal was to examine the impacts of gypsy moth defoliation on radial growth and carbon accumulation of major host species of gypsy moth in central region of Korea. The detailed objectives were (1) to evaluate the extent and duration of damage caused by gypsy moth defoliation and (2) to assess the ability to resist and recover from gypsy moth defoliation depending on host species. To do this, we studied two host species—*Larix kaempferi* and *Quercus acutissima*—in both defoliated and non-defoliated sites. Trees in the non-defoliated site served as a control against the potentially confounding effect of climate^25^. In central Korea, *L. kaempferi* is the one of the major plantation species and was the severely defoliated host in the recent outbreak of AGM^29^, while *Q. acutissima,* a moderate defoliated host, is a major component of secondary forests in urban forest areas and has the highest carbon absorption capacity among *Quercus* species in central Korea^28,30^.

## Results

### Changes in radial growth variables

The annual mean tree-ring width (TW) values before the gypsy moth outbreak and defoliation event of 2010–2019 were 1.34 ± 0.65 mm for *L. kaempferi* and 1.92 ± 0.80 mm for *Q. acutissima* at the defoliated site and 1.53 ± 0.64 mm and 2.64 ± 0.69 mm at the non-defoliated site (Table 1). Without the age-trend correction, *L. kaempferi* trees at the two sites had similar annual radial growth during the pre-event period. For *Q. acutissima*, latewood width (LW) and TW values were higher in the non-defoliated than the defoliated site, while BAI values showed no difference between two sites. In contrast to the pre-event period, the radial growth of *L. kaempferi* trees was much lower at the defoliated site than for trees at the non-defoliated site during the gypsy moth defoliation event and post-event period. However, *Q. acutissima* trees showed lower radial growth at the defoliated site during the year of gypsy moth defoliation, but not in subsequent years in the post-event period.

**Table 1:** Mean and standard deviation of radial growth variables at the pre-defoliation event period, during defoliation, and the post-event period. Significance of differences (*p* < 0.05) in radial growth between defoliated and non-defoliated sites was determined with Mann–Whitney U-tests, and significance is shown in bold. Abbreviations: EW, earlywood width; LW, latewood width; TW, tree-ring width; BAI, basal area increment.

Species	Period	Variable	Defoliated	Non-defoliated	U	*p*-value
*L. kaempferi*	Pre	EW (mm)	0.84 ± 0.38	0.98 ± 0.39	104	0.26
		LW (mm)	0.50 ± 0.29	0.55 ± 0.29	125	0.71
		TW (mm)	1.34 ± 0.65	1.53 ± 0.64	112	0.40
		BAI (cm^2^)	12.58 ± 7.38	13.80 ± 6.11	117	0.51
	During	EW (mm)	0.80 ± 0.35	1.11 ± 0.51	89	0.09
		LW (mm)	**0.08 ± 0.12**	**0.49 ± 0.29**	**9**	**0.00**
		TW (mm)	**0.88 ± 0.44**	**1.60 ± 0.78**	**54**	**0.00**
		BAI (cm^2^)	**8.68 ± 5.21**	**15.36 ± 8.10**	**58**	**0.00**
	Post	EW (mm)	**0.48 ± 0.29**	**1.17 ± 0.55**	**32**	**0.00**
		LW (mm)	**0.33 ± 0.19**	**0.89 ± 0.47**	**30.5**	**0.00**
		TW (mm)	**0.81 ± 0.46**	**2.05 ± 0.94**	**30**	**0.00**
		BAI (cm^2^)	**8.08 ± 5.54**	**20.18 ± 10.49**	**34**	**0.00**
*Q. acutissima*	Pre	EW (mm)	0.69 ± 0.16	0.83 ± 0.16	34.5	0.06
		LW (mm)	**1.23 ± 0.69**	**1.80 ± 0.61**	**28**	**0.02**
		TW (mm)	**1.92 ± 0.80**	**2.64 ± 0.69**	**30**	**0.03**
		BAI (cm^2^)	19.58 ± 9.21	22.93 ± 6.65	50	0.38
	During	EW (mm)	**0.62 ± 0.17**	**0.85 ± 0.19**	**22**	**0.01**
		LW (mm)	**0.43 ± 0.31**	**1.51 ± 0.56**	**4**	**0.00**
		TW (mm)	**1.04 ± 0.33**	**2.36 ± 0.68**	**6**	**0.00**
		BAI (cm^2^)	**11.50 ± 5.12**	**22.90 ± 7.64**	**14**	**0.00**
	Post	EW (mm)	0.74 ± 0.17	0.85 ± 0.21	40	0.13
		LW (mm)	1.46 ± 0.91	2.00 ± 0.75	45	0.23
		TW (mm)	2.20 ± 1.00	2.85 ± 0.86	38	0.10
		BAI (cm^2^)	23.99 ± 11.73	28.13 ± 9.48	52	0.45

**Figure 1:**
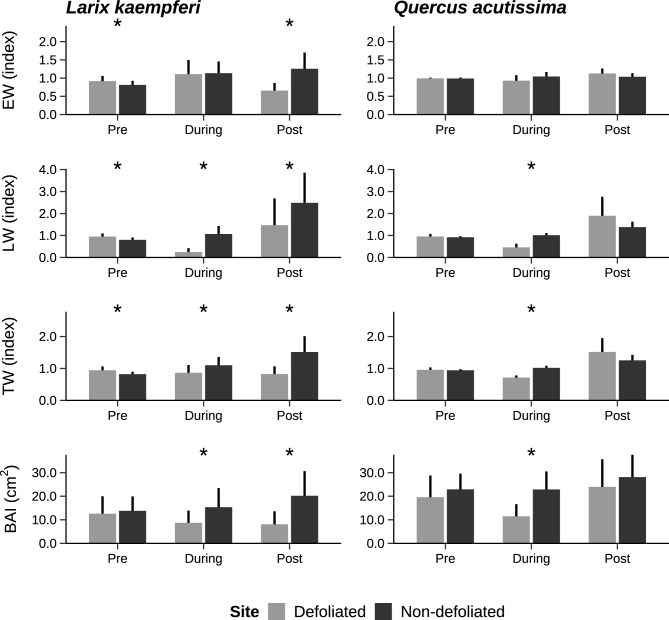
Ring width indices and basal area increments of *Larix kaempferi* and *Quercus acutissima* in the pre-defoliation, during defoliation, and post gypsy moth defoliation event of 2020. Error bars indicate standard deviations. Asterisks above the bars indicate significant differences between sites when tested by Mann–Whitney U-test (*p* < 0.05). Abbreviations: EW, earlywood width; LW, latewood width; TW, tree-ring width; BAI, basal area increment.

When considering the detrended ring width index, the results showed a clear pattern dependent on the tree species and the radial growth variables (Fig. 1). During the pre-event period, *L. kaempferi* trees at the defoliated site had significantly larger EW, LW and TW index values than at the non-defoliated site (*p* < 0.05), whereas for *Q. acutissima*, there were no differences in the ring width index values between two sites. However, when the gypsy moth defoliation occurred, for both species there were differences between the two sites for all ring width indexes except EW, with lower values of both trees at the defoliated site (*p* < 0.05). In years after the defoliation, *L. kaempferi* trees at the defoliated site continued to show lower values for the ring width index. In contrast, in the post-event period, *Q. acutissima* trees at the defoliated site showed no difference in radial growth from trees at the non-defoliated site. Significant differences between the two sites in basal area increment were observed in both the defoliation year and the post-event period for *L. kaempferi* trees, and for *Q. acutissima*, growth was reduced at the defoliated site only during the year of defoliation.

### Resistance, recovery, and resilience indices of radial growth variables

**Figure 2:**
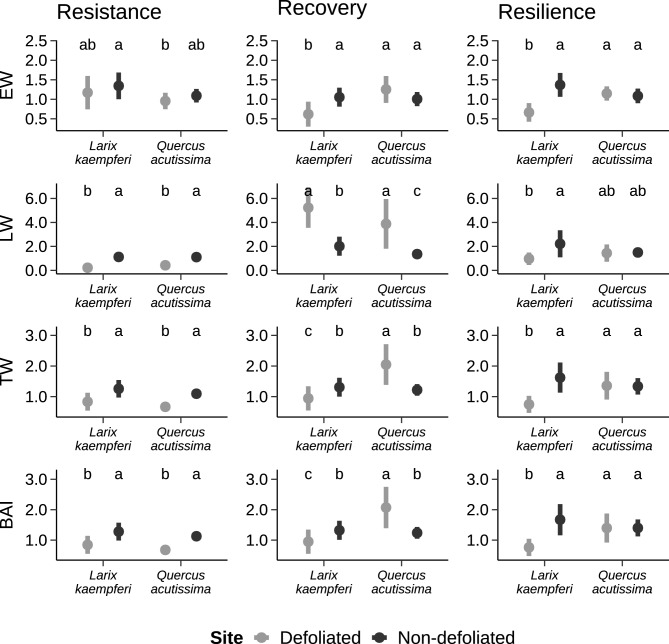
Resistance, recovery, and resilience indices for ring widths and basal area increments of *Larix kaempferi* and *Quercus acutissima* at sites that were either defoliated or not defoliated during the gypsy moth defoliation event of 2020. Different lowercase letters indicated the significant differences at the 0.05 level tested by two-way analysis of variance or Kruskal–Wallis test. Abbreviations: EW, earlywood width; LW, latewood width; TW, tree-ring width; BAI, basal area increment.

The resistance, recovery, and resilience indices, calculated from four radial growth variables, showed significant differences depending on the presence or absence of gypsy moth defoliation and on the tree species (Fig. 2). In both species, the resistance index based on BAI was consistently higher in the non-defoliated site (*p* < 0.05). High resistance index values for the non-defoliated site were also found for the EW, LW, and TW variables. However, there were no significant differences between species for any radial growth variables within the same site.

Recovery index values showed various patterns according to the tree species. *L. kaempferi* trees at the defoliated site had lower recovery index values of radial growth than that at non-defoliated site, except for the LW (*p* < 0.05). In contrast to *L. kaempferi*, for *Q. acutissima* the recovery indices for BAI, TW, and LW was higher at the defoliated site than at the non-defoliated site. The differences in recovery and resilience indices for the defoliation event were found to be larger for *L. kaempferi* than for *Q. acutissima.* Defoliated *L. kaempferi* trees had the lowest resilience index values, while for *Q. acutissima* the resilience index showed no difference between defoliated and non-defoliated trees.

### Individual-level carbon accumulation

**Figure 3:**
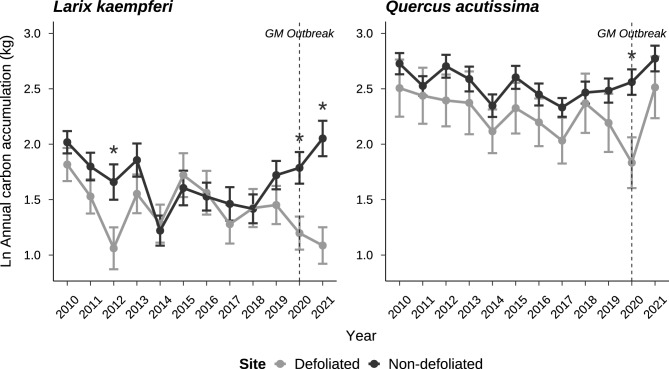
Annual carbon accumulation (kg) of *Larix kaempferi* and *Quercus acutissima* as affected in 2020 by gypsy moth defoliation. Points and error bars represent means and standard errors of individual-level annual carbon accumulation. Asterisks indicate significant differences (*p* < 0.05) as determined by repeated measure two-way ANOVA with Bonferroni correction.

As shown in the results of the pairwise comparisons, before the gypsy moth outbreak of 2020, there were no significant differences in the annual carbon accumulation between the defoliated and non-defoliated sites for either tree species except in the year 2012 for *L. kaempferi* (Fig. 3). In the pre-event period of 2010–2019, regardless of gypsy moth defoliation, *Q. acutissima* exhibited higher carbon accumulation than *L. kaempferi*. During this period, the average carbon accumulation absorbed annually by an individual tree was 5.8 ± 3.2 kg year^−1^ in *L. kaempferi* and 12.9 ± 5.3 kg year^−1^ in *Q. acutissima*. However, annual carbon accumulation for both species was significantly reduced at the defoliated site in the year of the gypsy moth outbreak (*p* < 0.001). In 2020, *L. kaempferi* and *Q. acutissima* trees at the defoliated site absorbed 4.0 ± 2.7 kg year^−1^ and 7.5 ± 3.9 kg year^−1^, respectively, which was about 42.5% and 46.1% less carbon than the trees at non-defoliated site (which were 6.9 ± 3.9 kg year^−1^ for *L. kaempferi* and 13.9 ± 5.2 kg year^−1^ for *Q. acutissima*). A reduction in carbon accumulation was also found the next year for *L. kaempferi* and when defoliated trees accumulated just 59.5% of the amount of carbon accumulated by non-defoliated trees.

## Discussion

This study detected detrimental impacts of gypsy moth defoliation on both *L. kaempferi* and *Q. acutissima*, which are major species in central region of Korea. Impacts were reduction of radial growth and decline in carbon accumulation during and after the outbreak year. For the intense defoliated host, *L. kaempferi,* defoliation caused prolonged suppression of radial growth with low resilience, while the moderated defoliated host, *Q. acutissima*, showed rapid recovery of radial growth after gypsy moth defoliation. This host-specific growth response was also associated with the ability to accumulate carbon. The reduction of carbon accumulation in defoliated trees was greater and lasted longer in *L. kaempferi*, and this impact will affect the ecological functioning of forests in central Korea during and after gypsy moth outbreaks.

In this study, *L. kaempferi* showed a prolonged depression of all radial growth variables until the year following gypsy moth defoliation, while *Q. acutissima* showed no growth depression of radial growth (Table 1). Some *Quercus* species are known to suffer growth declines for 2–4 years after gypsy moth defoliation^31^, however, no such lagged effects were observed in *Q. acutissima*. Damage caused by defoliation can vary depending on the characteristic of defoliation, especially its duration and severity^19^. In particular, the more preferred host species tend to be more severely damaged during episodes of weak or moderate defoliation^32^. In North America, *Quercus* species are preferred by EGM over other host species, making them susceptible to defoliation, and they also suffer from severe damage to growth after defoliation^33,34^. Red oak, *Q. rubra* (the most preferred host of EGM) also experiences the largest and longest depression of radial growth, followed by lesser effects for the intermediate and non-preferred hosts^9^. While feeding on non-preferred species may increase when favored resources are scarce due to the heavy or successive defoliation^32^. For example, *Abies balsamea* (L.) Mill., which is known to be a poor host for EGM in North America, actually suffered more damage than susceptible tree species during episodes of heavy forest defoliation in New Brunswick, Canada, losing an average of 55% of the wood volume^23^. The recent gypsy moth outbreak in Korea, which was a one-year event and was not intensive enough to cause tree mortality, is likely to have resulted in concentrated defoliation on preferred hosts rather than less preferred hosts. These less severe defoliation conditions may explain some of the observed differences in damage on the radial growth between *L. kaempferi* and *Q. acutissima*. However, in a laboratory feeding trials, Asian gypsy moth population showed the higher larval survival and performance in *Quercus velutina* Lam. than *Larix occidentalis* Nutt.^35^. Additional research on feeding preferences is necessary to clearly elucidate the relationship between host preference and the damage on the radial growth.

Another reason for differential growth response could be the biotic and abiotic factors related to the gypsy moth population density and the susceptibility of host species. The intensity of defoliation can be directly related to the density of the gypsy moth population^36^. In this study, both species were subjected to similar environmental conditions, suggesting that the impact of abiotic factors, such as climate and soil, on controlling the gypsy moth population was considered minimal. On the other hand, biological factors, particularly predation by small mammals, are likely to have played a significant role in regulating the gypsy moth population based on forest type^37^. The findings which found higher predation intensity and density of small mammals in oak forests compared to *L. kaempferi* forests, may explain why defoliation severity was higher in *L. kaempferi* stands than *Q. acutissima* in this study^10^. Meanwhile, from the perspective of the susceptibility of host species, the mean sensitivity of tree-ring series was lower for *Q. acutissima* than for *L. kaempferi* (Table 2), which means that the current habitat has fewer factors limiting the radial growth of *Q. acutissima* trees, resulting in less annual variation in tree-ring width. Given the more constraints imposed by environmental factors on radial growth of *L. kaempferi*, its defensive capacity is expected to be limited^38^. Consequently, it is more likely to be susceptible to gypsy moth outbreaks, resulting in substantial radial growth loss when compared to *Q. acutissima*.

**Table 2:** Descriptive statistics of site chronologies during the common period of 1990–2021.

Species	Site	Core/tree	DBH (cm)	Inter-series correlation	Mean sensitivity	Rbar	EPS
*L. kaempferi*	Defoliated	17/17	31.5 ± 4.6	0.47	0.29	0.27	0.87
	Non-defoliated	16/16	31.9 ± 3.9	0.60	0.26	0.42	0.92
*Q. acutissima*	Defoliated	10/10	35.6 ± 9.8	0.47	0.19	0.34	0.83
	Non-defoliated	13/13	32.6 ± 4.8	0.68	0.16	0.53	0.93

During the gypsy moth defoliation in Korea, the biggest change in radial growth was founded in the latewood width (LW) for both species (Fig. 1), as seen in previous studies^20,39,40^. A plausible reason for this is the temporal overlap of the latewood formation with the gypsy moth larval feeding. Gypsy moth eggs hatch in central Korea in mid-April, and larvae vigorously feed on the leaves from June to July, causing a shortage of current photosynthate as a result of defoliation^27^. In the study region, latewood formation starts in late-May for *Quercus* species and in mid-June for *L*. *kaempferi*^41,42^. Extensive herbivory in these time periods may limit the formation of latewood by reducing the current photosynthate, which is a more important source for latewood formation than for earlywood^43^. Moreover, the secondary leaf flush that was observed in August after defoliation might be another reason for the reduction of LW because leaf replacement would consume current photosynthate for both species.

In contrast, we observed a relatively small impact of gypsy moth defoliation on EW, which may occur because earlywood cells are formed before the period of gypsy moth feeding. In general, many temperate deciduous species begin cambium reactivation (leading to early wood formation) in spring before the flushing of new leaves^44^. For example, ring-porous *Quercus* species begin the formation of earlywood vessels as early as mid-March using the photosynthate from the previous summer and autumn^45,46^. Similarly, in central Korea the deciduous conifer, *L*. *kaempferi* initiates cambium activity in mid-April and begins to form earlywood tracheids^42^. Since in most deciduous trees, earlywood formation is a product of both previous and current photosynthate^43,47^, the presence of pre-formed earlywood (synthesized with previous year photosynthate reserves) appears to offset the impact on earlywood of defoliation by using stored photosynthate to compensate for the shortage of current photosynthate, in both study species.

Except for earlywood width (EW), defoliated trees of both species were less resistant to gypsy moth defoliation than non-defoliated trees, showing growth reduction during the year of defoliation for all radial growth variables (resistance < 1). In general, stressed trees are likely to be damaged first^48^. Stressful conditions such as soil compaction or pollutants at the defoliated site (which was surrounded by an urbanized area), seem to have affected the stand vigor, making trees more susceptible to defoliation^49^. For four radial growth variables measured, the latewood of defoliated trees showed narrower (lower width), lower resilience, lower resistance, but higher recovery following gypsy moth defoliation (Fig. 2). These findings suggest that latewood characteristics are the most informative measures for detecting impacts on the radial growth response of trees to gypsy moth defoliation and for reconstructing past outbreaks^40,50^.

Defoliated *Q. acutissima* trees showed a rate of high recovery for all growth variables in the year following defoliation (Fig. 2). This may be in part explained by the compensatory growth habit^20^. *Quercus* species exhibit compensatory growth after damage from herbivory and can return to normal growth rates immediately after defoliation^51,52^. Defoliated *Q. serrata* Murray and *Q. crispula* Vuk. reduce their investment in shoots while increasing the number of leaves during subsequence flushes^53^. Moreover, the number of flushes increases after a defoliation event^53^. The rapid recovery in *Q. acutissima* may be the result of the same compensatory strategy against herbivory as seen in the above *Quercus* species. This is likely because *Q. acutissima* exhibits an indeterminate growth habit, with secondary leaf flushing occurring after defoliation^54^. Although *L. kaempferi* also has compensatory responses, such as increasing the rate of photosynthesis by the remaining leaves^55^, rapid growth recovery after defoliation was not observed in this study for this species. Whether this was influenced by the severity of the defoliation requires further research.

Species such as ring-porous *Quercus* and the deciduous conifers of the genus *Larix* are known to have lower declines in growth and lower mortality rates following defoliation than do the diffuse-porous trees or evergreen conifers^56^. Therefore, to comprehensively assess the impacts of gypsy moth defoliation, it would be necessary to investigate impacts on a broader range of hosts. Also, climate change will affect the host preferences of gypsy moth by altering the tree host’s physiological traits^57^. Investigating the host-specific growth responses of more host species should help clarify the relationship between host preference and damage following gypsy moth defoliation.

In the defoliation year, a significant decrease in the carbon accumulation was observed in defoliated trees for both species (Fig. 3). Defoliation not only reduces total carbon assimilation but also changes the allocation priority within the carbon pool^58^. Following defoliation, trees shift carbon allocation from vegetative growth to storage as non-structural carbohydrates (NSC) causing woody biomass accumulation significantly decrease under conditions of insufficient carbon uptake^58,59^. This pattern of reallocation explains the subsequent decline of radial growth in defoliated *L. kaempferi*. In *Larix gmelinii* (Rupr.) Rupr., 43% of carbon in starch pool for wood formation of the current year came from old C storage^60^. So, declines in carbon assimilation in a defoliation year may result in a reduction of the radial growth in the following year.

Furthermore, the severity of defoliation determines the levels of carbon accumulation and mortality. As the severity of defoliation increases, carbon allocation to wood gradually decreases^61^. Moreover, reductions in total NSC due to severe defoliation leads to carbon depletion and increased risk of tree mortality^62^. To survive under carbon-limiting conditions, trees have to maintain a certain amount of NSC. If the NSC storage falls below a certain threshold, the risk of mortality is likely to increase^62^. In this study, the cumulative reduction in carbon assimilation was greater in the severely defoliated host *L. kaempferi*. This species is more likely to suffer carbon starvation and increased mortality than moderated defoliated host *Q. acutissima*. Therefore careful attention needs to be paid to the impact of such defoliation on carbon storage and the consequences for sustaining forest functions. For adaptive forest management, improving the individual tree’s vigor and mixing stands with resilient species should minimize the impacts of defoliation^63,64^. Silvicultural treatment such as thinning may also reduce forest susceptibility to defoliation-induced mortality and promote rapid recovery after defoliation events^65^.

## Conclusions

Future bouts of gypsy moth defoliation will likely affect forest function by reducing radial growth and carbon assimilation, with the extent of the impact varying based on the defoliation severity depending on host species. This suggests that adaptive forest management should take into consideration the host-specific growth responses of tree species in order to maximize overall forest resilience to gypsy moth defoliation. Future studies need to examine more hosts species and clarify the effect of host preference on the impacts of defoliation by gypsy moth.

## Materials and methods

### Study site

**Figure 4:**
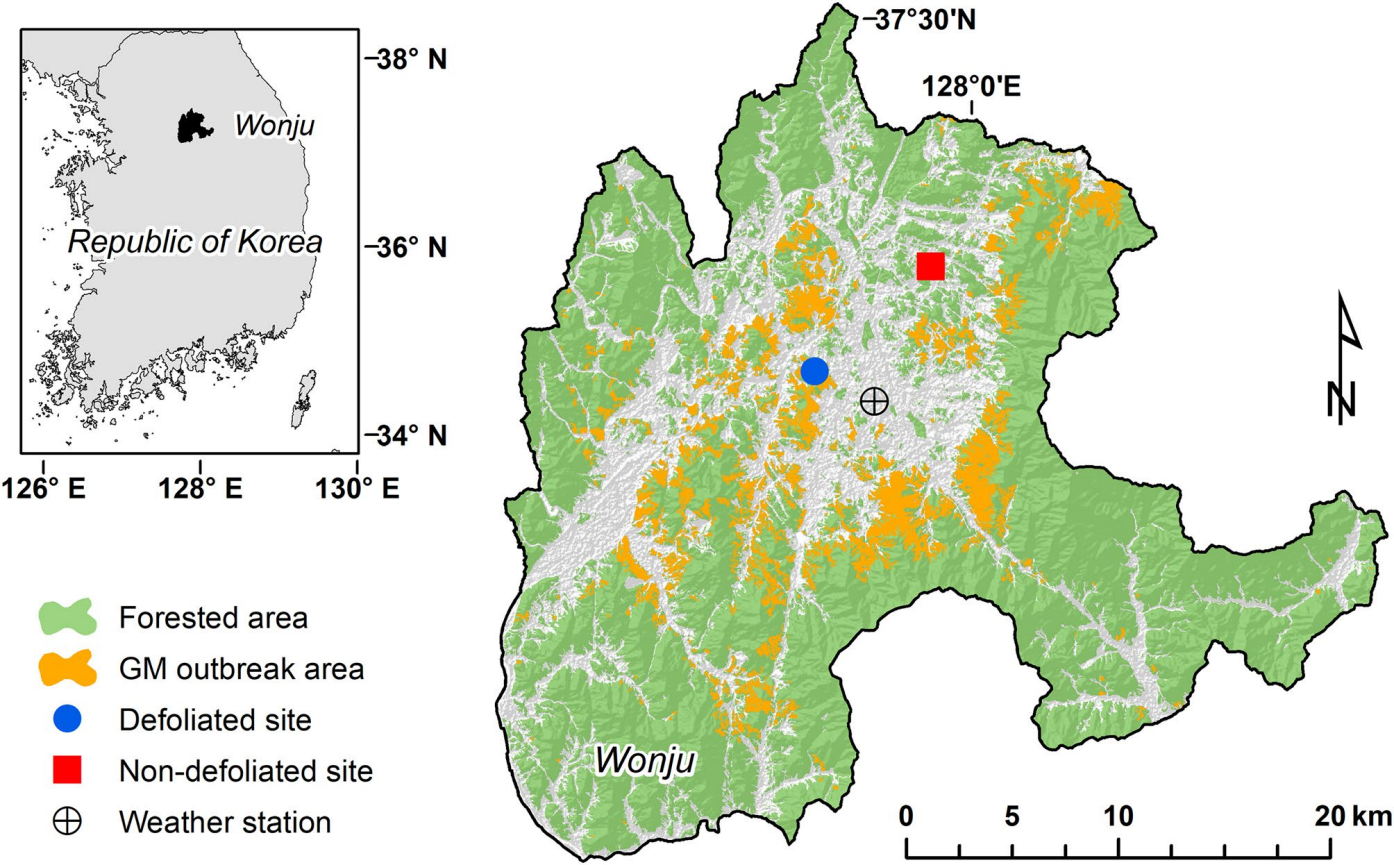
Location of study sites. The area with gypsy moth outbreaks (yellow) is modified from Choi et al.^29^. The blue circle indicates the defoliated site where gypsy moth (GM) was in outbreak in the summer of 2020. The red square indicates the non-defoliated site in this study. The black cross-circle indicates the location of the nearest weather station, from which all climatic data were obtained. Map was created using ArcGIS Desktop 10.2 (https://www.esri.com/en-us/arcgis/products/arcgis-pro/overview).

This study was conducted in Wonju city (37° 20ʹ N, 127° 55ʹ E) in the central part of Korean peninsula (Fig. 4). The region has a temperate climate with dry winters and humid summers. Data obtained from the nearest weather station showed a mean annual temperature for 1992–2021 of 12.0 ℃, ranging from 3.1 ℃ in January and 25.4 ℃ in August^66^. Mean annual precipitation over the same period was 1293 mm, and 60% of the total precipitation fell between June and August in the summer season. Following unusually high winter temperatures and spring drought in 2020, gypsy moth populations dramatically increased in the area, causing serious damage to crown of multiple hosts during the mid-summer^28^. Among the defoliated hosts, *L. kaempferi* experienced the most significant damage from this gypsy moth outbreak compared to other host species. The gypsy moth damaged 22% of all the *L. kaempferi* plantations in the study area, and 274 ha out of total 5306 ha of larch plantations were severely defoliated^29^. The large gypsy moth outbreak in 2020 was thought to be a unique event, from only a single year. The gypsy moth nuclear polyhedrosis virus (NPV) and its parasitoids increased during the outbreak, and by the following year (2021) the population density of gypsy moth collapsed^13,67^.

In November of 2021, we chose two forested areas with *L. kaempferi* plantations and adjacent stands of *Q. acutissima*, one area that was defoliated in 2020, while the other site (the control) was chosen from a similar area that had not been previously defoliated^29^. Both sites were located on gentle slopes of 15°–25°, with the defoliated site facing southwest (262 m above the sea level) and the non-defoliated site facing northeast (181 m above the sea level). The larch plantations and *Q. acutissima* stands were 31–40 years old and they were located within 300 m of each other within the same site. Before the sampling, the designation as defoliated or non-defoliated (in the previous year) was confirmed by a visual check of gypsy moth egg mass numbers at the two sites. The site defoliated in 2020 was located in a small forest stand near an urbanized area, and traces remained on tree trunks of large numbers of egg masses. No sign, however, was seen of increased tree mortality in 2021 (the year of our observations). In contrast, the non-defoliated site, which was located in a forested area about 7.5 km northwest of the defoliated site, had no-signs of large numbers of egg masses.

### Tree coring and measurement of radial growth

For core sampling, 10–17 dominant or co-dominant trees in each site were selected of each of two species (*L. kaempferi* and *Q. acutissima*), and their diameter at breast height (DBH, 1.3 m above the ground) was measured. One core per each tree was extracted at this height using a 5.15 mm increment borer (Haglöf Sweden AB, Långsele, Sweden) while avoiding taking cores on the side of slope. Sample cores were stored in plastic straws and taken to the laboratory for analysis. Following standard dendrochronological methods, the cores were air-dried and glued onto wooden mounts. Then, using sandpaper with up to 400 grit, cores were sanded until the surface was clearly visible^68^. Under the stereomicroscope (Stemi 305, Carl Zeiss, Oberkochen, Germany), cross-dating between cores was conducted using the list method^69^. Cross-dated cores were scanned at 2400 dpi with an EPSON V370 scanner (Epson Corp., Nagano, Japan) and the earlywood width (EW), latewood width (LW) and tree-ring width (TW) were measured to 0.001 mm precision with a WinDENDRO 2016a (Regent Instruments, Quebec, Canada). The quality of cross-dating was evaluated using COFECHA software^70^.

For calculating basal area increments (BAI), which is an accurate measure of production, annual diameters were reconstructed using the current measured DBH and annual tree-ring widths, and then converted to BAI using the following formula.

$${\text{BAI}}_{t} = \pi \times \left( {\left[ {{\text{DBH}}_{t} /2} \right]^{2} \left[ {{\text{DBH}}_{t - 1} /2} \right]^{2} } \right)$$

where BAI_*t*_ is annual basal area increment (cm^2^) of corresponding year *t* and defined as the difference of basal area at two consecutive points in year (*t* and *t *− *1*). DBH (cm) is the reconstructed diameter at breast height under the assumption of circular growth in each year.

### Chronology building

**Figure 5:**
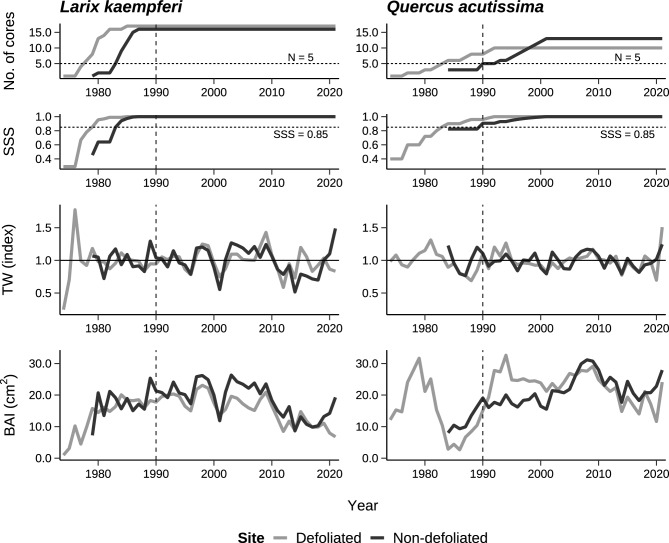
Site chronologies of the tree-ring width index and the basal area increment (cm^2^) of two species, *Larix kaempferi* and *Quercus acutissima*. The vertical dashed line indicates the first year of common period of 1990–2021.

Chronologies were built from three ring width series (and consequent basal area increment) for each tree species and study site using dplR package in R program^71,72^. To remove any age or size-related trends, the ring width series were standardized with negative exponential curves or spline curves of 50% frequency response at a wavelength of 2/3^73^. Each detrended ring width series and basal area increment was averaged with a bi-weight robust mean and upscaled to the site-level chronologies. Then, all chronologies were truncated at the year 1990 where the subsample signal strength (SSS) reaches the threshold value 0.85^74^ and the number of samples was greater than five (Fig. 5). Basic statistics, including inter-series correlation, mean sensitivity, Rbar, and expressed population signal (EPS), were calculated^75^ for the common period of 1990–2021 (Table 2).

### Estimation of carbon accumulation from above-ground biomass

Annual carbon accumulation was estimated from the above-ground biomass (AGB) assuming a constant carbon concentration in all tissues. To estimate the individual-level AGB from sampled trees, simplified allometric equations were employed for each species^76^. Based on the estimated annual AGB, annual carbon accumulation was calculated by multiplying species-specific carbon conversion factor as followings.

$$\begin{aligned} & {\text{AGB}}_{t} = a \times {\text{DBH}}_{t}^{b} \\ & {\text{AGBI}}_{t} = {\text{AGB}}_{t} {-}{\text{AGB}}_{t - 1} \\ & {\text{Carbon}}\;{\text{accumulation}} = cf \times {\text{AGBI}}_{t} \\ \end{aligned}$$

where AGB_*t*_ is the above-ground biomass of an individual tree (g) and DBH_*t*_ is the reconstructed annual diameter (cm) of year *t*. The two parameters *a* and *b* are species-specific constants obtained from previous study^76^, namely 44.49 and 2.70 for the *L. kaempferi* (R^2^ = 0.96) and 143.38 and 2.49 for *Q. acutissima* (R^2^ = 0.94), respectively. AGBI_*t*_ is annual AGB increment, calculated as the difference of AGB between two consecutive years (*t* and *t − 1*). A parameter *cf* is carbon conversion factor of 0.50 for *L. kaempferi* and 0.48 for *Q. acutissima*^76^.

### Resilience indices

To evaluate the capacity of the radial growth to withstand the gypsy moth defoliation, three growth-based resilience indices were calculated^77^. Resistance is defined as the ability to retain the growth through the disturbance event, and it is calculated as the ratio of the growth during the gypsy moth defoliation to pre-event growth in this study. Recovery indicates how much a tree recovers the growth lost from damage after the disturbance ends. To calculate the recovery index, the post-event growth was divided by the growth during the defoliation. Resilience is defined as the ability to sustain the growth rate present that existed before to a disturbance once the disturbance has passed and was calculated as the ratio of post-event growth to pre-event growth using the following formulae^77^.

$$\begin{aligned} & {\text{Resistance}}\left( {{\text{Rt}}} \right) = {\text{G/Pre-G}}, \\ & {\text{Recovery}}\left( {{\text{Rc}}} \right) = {\text{Post-G/G}} \\ & {\text{Resilience}}\left( {{\text{Rs}}} \right) = {\text{Resistance}} \times {\text{Recovery}} = {\text{Post-G/Pre-G}} \\ \end{aligned}$$

where G is the radial growth during the gypsy moth defoliation of 2020. Pre-G indicates the average radial growth of the 3 years preceding the defoliation year. Due to lack of growth in the year after the defoliation event, Post-G is defined only as the radial growth of the first year following the defoliation year in this study. All resistance, recovery, and resilience indices were calculated at the individual tree level using each ring width series and basal area increments, which were then averaged to obtain site level values for comparison.

### Statistical analysis

Differences in radial growth between defoliated and non-defoliated trees were investigated in three time periods—pre-event, during the event, and post-event—using the Mann–Whitney U-test. Radial growth in the pre-event period was characterized using three measures, the annual mean of the raw ring widths, the ring width index, and the basal area increment from 2010 to 2019. The raw ring width, ring width index and basal area increment of the defoliation year in 2020 and that of one year after the event in 2021 were used as measures of the radial growth during the defoliation period and post-event period, respectively.

Two-way analysis of variance (ANOVA) and post-hoc Tukey's test were applied to test effects of species and defoliation event (site) on resilience indices for each radial growth variables. When normality or equal-variance assumptions were not satisfied, the non-parametric Kruskal–Wallis test was used. Multiple comparisons were made using Fisher's least significant difference (LSD) test with the Bonferroni correction.

The differences in annual carbon accumulation from the defoliation event (site) were assessed by use of repeated measures two-way ANOVA with Bonferroni correction for each species. For the period of 2010–2021, the analysis was performed using year as within-subjects variable and defoliation event (site) as between-subjects variable. To meet the assumption of normality, the outcome variable (carbon accumulation) was log-transformed before the analysis and examined for normality using Shapiro–Wilk test. The homogeneity of variance across between-subjects was tested by Levene’s test and the equality of variances of the differences between within-subjects was checked with Mauchly’s test of sphericity. All statistical analysis was conducted using R program^72^.

## Data Availability

The data from the current study are available from the corresponding author upon reasonable request.
